# Corticospinal excitability during observation of basketball free-throw movement: Effects of video playback speed and stimulus timing

**DOI:** 10.1371/journal.pone.0292060

**Published:** 2023-09-28

**Authors:** Masaya Kitamura, Katsuya Yamamoto, Atsushi Oshima, Kiyotaka Kamibayashi

**Affiliations:** 1 Graduate School of Health and Sports Science, Doshisha University, Kyotanabe, Kyoto, Japan; 2 Research Fellow of the Japan Society for the Promotion of Science, Chiyoda-ku, Tokyo, Japan; 3 Faculty of Health and Sports Science, Doshisha University, Kyotanabe, Kyoto, Japan; Universita degli Studi di Torino, ITALY

## Abstract

Transcranial magnetic stimulation studies have indicated that action observation (AO) modulates corticospinal excitability. Although a few previous studies have shown that the AO of simple motor movements at a slow playback speed facilitates corticospinal excitability more than that at normal playback speed, it is unclear if this effect occurs during the AO of sport-related complex movements. Therefore, we investigated the changes in the motor evoked potential (MEP) amplitudes of the flexor carpi radialis (FCR) and abductor digiti minimi (ADM) muscles during the AO of a basketball free-throw movement at three different playback speeds (100%, 75%, and 50% speeds). Additionally, we evaluated the effects of stimulus timing (holding the ball vs. releasing the ball for shooting) and motor expertise (expert basketball players vs. novices) on the MEP amplitude during the AO. Our results demonstrated that regardless of motor expertise, the MEP amplitude of the FCR muscle was significantly smaller in the 50% speed condition than in the 100% condition. In the ADM muscle, the MEP amplitude was significantly larger when the ball was held after dribbling than when the ball was released. Therefore, it is suggested that corticospinal excitability in specific muscles during the observation of complex whole-body movements is influenced by video playback speed and stimulus timing.

## Introduction

The observation of the motor behavior of other people is supposed to cause the observer to internally simulate the observed motor action [1]. This internal motor simulation during action observation (AO) has been suggested to improve motor performance and learning [2–4]. The neural mechanism underlying AO-induced motor learning is considered to involve functional plastic changes in the primary motor cortex (M1) [5] and primary somatosensory cortex [6].

Brain activation during AO has been intensively investigated since the discovery of the firing of “mirror neurons” in area F5 of a monkey’s premotor cortex during AO [7–9]. These neurons were found to be activated both when the monkey executed an action and when the monkey observed a similar action performed by another monkey or the experimenter [8]. Consistent with these studies, brain imaging studies in humans have shown that AO induces activity in the motor-related brain areas [10,11]. In humans, this system is called the “mirror neuron system” (MNS) [12], which includes the inferior parietal lobule, ventral premotor cortex, and caudal part of the inferior frontal gyrus [13]. In addition to brain imaging analysis, transcranial magnetic stimulation (TMS) is another widely used method to investigate the MNS activity. In TMS, a single TMS pulse is delivered to M1 to elicit an electromyographic (EMG) response in the targeted muscle. The amplitude of the response called motor evoked potential (MEP) represents the corticospinal excitability at the stimulus timing. Numerous reports have shown that MEP amplitude increases during AO compared to that during rest or observation of a static image [14–19], specifically in the muscles activated while performing an observed action [14,15]. In addition, the MEP amplitude during AO changes according to the phase of the observed action [20,21]. A previous study [21] applied TMS at several phases of the movement during observation of a reaching-and-grasping movement reported that the largest MEP amplitude of the first dorsal interosseous muscle was recorded at the hand-closing phase, indicating that corticospinal excitability during AO is the most facilitated during the phase in which that muscle would be activated during the execution of that movement. These reports imply that during AO, corticospinal excitability is affected through activation in the MNS [14,15,20,21].

Corticospinal excitability has also been shown to be modulated by video playback speed during AO [22,23]. Moriuchi et al. [22] demonstrated that the MEP amplitudes of the thenar and abductor digiti minimi (ADM) muscles during the AO of ball catching with a bare hand were larger in the slow playback speed condition than in the normal speed condition. They suggested that participants could recognize the elements of the action more easily at the slow video playback speed [22]. However, the same research group reported that video playback speed did not affect the MEP amplitude when the reaching movement to lift a ball was observed [23]. On the other hand, a study examining the effect of the actual speed in the observed movement showed that when compared to a static image condition, corticospinal excitability was facilitated during the AO of complex finger motor sequences at 2 Hz but not at 1 Hz [24]. Thus, the effect of the speed of the observed movement on corticospinal excitability remains unclear, which may vary depending on the type of movement.

Videos with slow playback speed are extensively used to evaluate the movement form or learn novel movements in sports practice. Although the effect of the observed movement speed on corticospinal excitability during AO was investigated, the observed actions were only movements of fingers [22–24]. Since physical movements in sports are more complex, involving numerous muscles in the whole body, it is important to verify the effect of slowing down the video playback speed of a complex movement on the excitability of the motor system during AO. Therefore, this study aimed to investigate the effect of decreasing playback speed on corticospinal excitability when observing a video of a basketball free throw, an action that involves the whole body, requiring a kinetic chain from the lower to the upper limbs. A previous study [24] revealed that while participants were observing a complex finger motor sequence at 2 Hz (a rate of execution similar to the individual spontaneous one), MEP amplitudes were significantly larger than those measured during the observation of the static hand. On the contrary, MEPs during the observation of the 1 Hz video were not significantly different from those during the observation of the static hand. Therefore, we hypothesized that when compared with a normal playback speed, a decrease in the video playback speed of the free-throw movement would reduce the corticospinal excitability. In addition, to investigate whether the effect of TMS timing during AO on corticospinal excitability differs among muscles, we recorded the MEP amplitude of the flexor carpi radialis (FCR) and ADM, as they are the main muscles activated during a free-throw movement when releasing and holding the ball, respectively. Moreover, we hypothesized that the MEP amplitudes in the FCR and ADM muscles would increase at the respective phases in which each muscle would be activated when executing the free-throw action because the corticospinal excitability during AO is the most facilitated at the phase of movement in which that muscle is activated during the execution of the observed action [20,21]. Furthermore, we compared the MEP responses during AO between experts and novices because a previous study had shown that the MNS was strongly activated in experts when they observed motor tasks within their motor repertoire [25]. The results of this study will provide new insights from a neurophysiological aspect regarding the use of AO in sports.

## Materials and methods

### Participants

Nine expert basketball players (mean age: 18.9 years, standard deviation [SD]: 0.7 years, basketball experience: >6 years) and nine basketball novices (mean age: 22.2 years, SD: 1.4 years) were included. Participants were recruited between September and November 2020. Experts were recruited from a university basketball team, and most of them competed in the Japanese National Championships during high school. Novices were students who had played basketball only in their physical education classes during school. All participants were male and self-reported as right-handers. The study was approved by the Ethical Committee of Doshisha University on Human Subject Research (approval no. 19052), and all participants provided written informed consent. The experiment was conducted in accordance with the Declaration of Helsinki.

### Video clips

**Fig 1:**
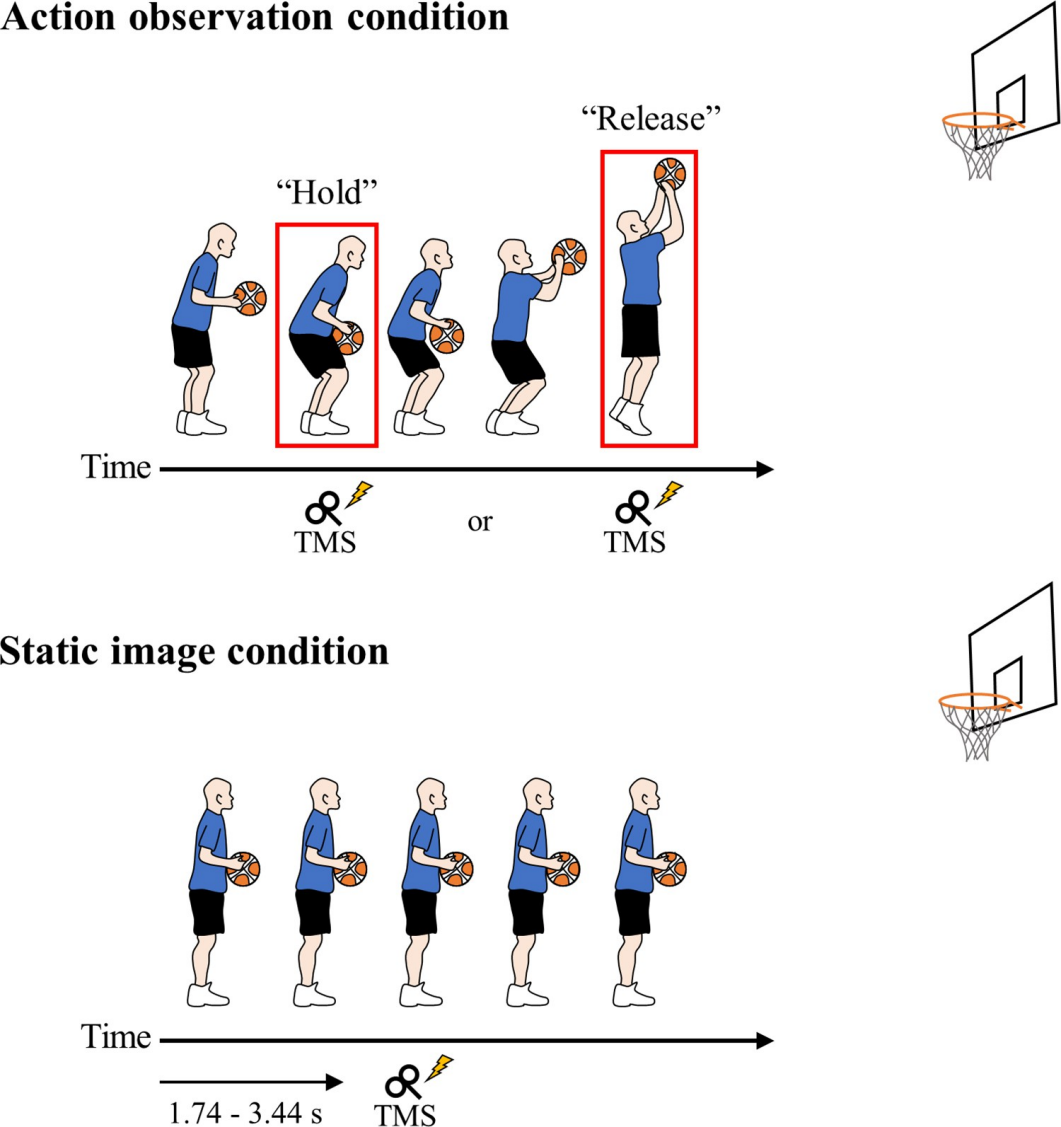
Illustration of the observation conditions and transcranial magnetic stimulation (TMS) timings. Participants observed videos of a basketball free-throw movement played at three different speeds (100%, 75%, and 50% playback speeds). The experiment consisted of two conditions: the action observation (AO) condition and the static image condition (a static image of a player standing still while holding the ball). In the AO condition, a TMS pulse was delivered at the time when the ball was held immediately after dribbling with a flexed posture of the three lower limb joints (“Hold”) or at the time when the ball was released (“Release”). Each rectangle depicted in each image indicates TMS timing. In the static image condition, the TMS pulse was applied between 1.74 and 3.44 s after the start of the image presentation. The individual in this figure has given written informed consent (as outlined in the PLOS consent form) to publish these illustrations.

Free-throw movements by two right-handed male basketball players from the same university basketball team were recorded using a digital camera (HDR-CX680, SONY, Japan) from the right side of each player (Fig 1). One successful free-throw attempt of each player was selected for video observation (two clips of the free-throw movement in total). The scenes from the start of the player’s routine dribble to the ball passing through the hoop were clipped (approximately 4.5 s). Subsequently, the playback speed of the videos was edited to show the movement at 100% (normal speed), 75%, and 50% speeds using video editing software (EDIUS NEO 2.5, Grass Valley, Japan). The control condition consisted of static images of the player standing still while holding a basketball (static image condition, approximately 4.0 s presentation) (Fig 1).

### EMG recording and TMS

EMG recordings of the FCR and ADM muscles of the right hand were obtained using pairs of adhesive Ag-AgCl surface electrodes (F-150S, Nihon Kohden, Japan). A ground electrode was placed on the ulnar styloid process. The EMG signals were amplified (gain = 1000) with a bandwidth frequency ranging from 5 Hz to 1 kHz using an amplifier (MEG-6108, Nihon Kohden). The signals were A/D converted at a sampling frequency of 2 kHz (PowerLab 16/35, ADInstruments, Australia) and analyzed on a PC using software (LabChart 8, ADInstruments).

The TMS pulse was delivered via a 70 mm outer diameter figure-of-eight coil connected to Magstim 200^2^ (Magstim, Whitland, UK). At the start of the experiment, we searched for the optimal stimulus locations on the left hemisphere where MEPs were detected in the right FCR and ADM muscles. The coil was placed tangentially to the scalp with the handle pointing backward and rotated 45° from the mid-sagittal line. The coil position was tracked during the MEP recordings using a TMS navigation system (Acuity Inc., Japan) to maintain the same coil position. Resting motor threshold (rMT) was defined as the lowest intensity required to evoke a MEP with an amplitude of >50 μV in at least 5 out of 10 stimuli in the FCR muscle. The test stimulus intensity was set at 120% rMT. In this study, the stimulation intensity ranged from 61% to 74% of the maximum stimulator output in experts (mean: 65.2%, SD: 4.7%) and 50–83% in novices (mean: 60.1%, SD: 11.4%).

### Experimental procedure

Participants on a reclining chair 80 cm away from a 50-inch monitor (LC-50W35, SHARP, Japan) were instructed to relax their forearm and finger muscles in a pronated forearm position and to observe the video attentively. Subsequently, they observed free-throw videos played at three different speeds (100%, 75%, and 50% speeds) and the static image. TMS was applied at either the stimulus timing when the player in the video clips holds the ball immediately after dribbling with a flexed posture of the three lower limb joints, or when the player releases the ball, defining them as “Hold” and “Release,” respectively (AO condition in Fig 1). In the static image condition (Fig 1), the TMS was delivered at 1.74–3.44 s after the start of the image presentation. The protocol for the MEP recording was composed of five sessions. Each session consisted of three video playback speed conditions and one static image condition. Each video playback speed in the AO condition was presented four times per session, while the static image condition was presented twice per session (14 stimuli per session). In each session, TMS was delivered twice for each Hold and Release timing at each playback speed condition of the AO conditions. The order of the AO and static image conditions presented in each session and stimulus timing were randomized. Before each video clip was played, information about the condition (playback speed or static image) was provided to the participant by text on the monitor (i.e., “100%,” “75%,” “50%,” or “static image”). An interval of >2 s was provided between the end of the clip playback and the start of the subsequent clip playback. The number of TMS pulses was 20 in each video playback speed condition (10 in each stimulus timing) and 10 in the static image condition across the five sessions (a total of 70 MEPs for each participant).

### Data analysis and statistics

To estimate corticospinal excitability, the peak-to-peak amplitude of the MEP was calculated using EMG data. The root mean square of the EMG signal for 50 ms before the stimulation (−60 to −10 ms) was also calculated as the background EMG (BEMG) level. Firstly, if the BEMG value exceeded 10 μV for each stimulation, we determined that the muscle was not at rest at the timing of stimulation, and excluded the data of the BEMG and induced MEP in the muscle on the trial from the analysis. Subsequently, the mean and SD of the BEMG level of the remaining data were calculated for each muscle in each participant. Trials with a BEMG value larger than the mean + 3 SD were excluded. Furthermore, after the calculation of the mean and SD in the MEP amplitude, trials with MEP amplitude exceeding mean + 3 SD as outliers were excluded from further analysis [26]. Following these criteria, 6.3% and 4.6% of the whole data in the FCR and ADM muscles, respectively, were removed. To test whether the BEMG level changed across conditions, linear mixed-effects models were used. The fixed effects were group (two groups: expert and novice) and condition (seven conditions: static image condition and six AO conditions consisting of 3 playback speeds × 2 stimulus timings). Participants were modeled as random effects.

To test group differences between experts and novices in the MEP amplitude for the static image condition, an independent-samples t-test was performed for each muscle. Subsequently, MEP amplitudes recorded from each muscle were normalized using the z-transformation for each participant [14–17,27,28]. To examine the effects of motor experience, stimulus timing, and video playback speed on corticospinal excitability during AO, the mean normalized MEP amplitude in the static image condition was subtracted from that in each AO condition [28]. The effects of group, stimulus timing, and video playback speed on the normalized MEP amplitude were evaluated using linear mixed-effects models in each muscle. Fixed effects were group (expert and novice), stimulus timing (Hold and Release), and video playback speed (100%, 75%, and 50%). For post-hoc comparisons, multiple comparisons with Sidak’s correction were used. All statistical analyses were performed using SPSS (Version 29.0, IBM, USA). Statistical significance was set at *p* < 0.05. Data are presented as mean ± standard error.

## Results

### Background EMG

Table 1 shows the values of the BEMG level in each observation condition. The linear mixed-effects model did not show any significant fixed effects of group and condition or interaction of group and condition on the BEMG levels of the FCR [group: *F* (1,16) = 0.200, *p* = 0.660, condition: *F* (6, 96) = 1.487, *p* = 0.191, group × condition: *F* (6, 96) = 1.637, *p* = 0.145] or ADM muscles [group: *F* (1, 16) = 1.306, *p* = 0.270, condition: *F* (6, 96) = 1.931, *p* = 0.083, group × condition: *F* (6, 96) = 1.857, *p* = 0.096]. The mean and SD of the BEMG level in each observation condition for each participant are shown in S1 Table.

**Table 1:** Mean activity level of background electromyography in each observation condition for each group (Mean ± Standard error).

Muscle	Group	Observation condition						
		Static	Hold			Release		
			100%	75%	50%	100%	75%	50%
**FCR** **(μV)**	**Expert**	**3.30 ± 0.42**	**3.27 ± 0.39**	**3.08 ± 0.37**	**3.16 ± 0.37**	**3.30 ± 0.41**	**3.36 ± 0.39**	**2.98 ± 0.37**
	**Novice**	**3.19 ± 0.22**	**3.00 ± 0.21**	**3.16 ± 0.26**	**2.84 ± 0.19**	**3.16 ± 0.22**	**2.77 ± 0.15**	**2.95 ± 0.19**
**ADM** **(μV)**	**Expert**	**3.74 ± 0.48**	**4.06 ± 0.52**	**3.80 ± 0.45**	**4.18 ± 0.43**	**3.65 ± 0.37**	**3.64 ± 0.47**	**3.32 ± 0.40**
	**Novice**	**2.97 ± 0.29**	**3.38 ± 0.30**	**3.27 ± 0.30**	**3.06 ± 0.40**	**3.13 ± 0.36**	**2.95 ± 0.26**	**3.28 ± 0.37**

FCR: Flexor carpi radialis muscle, ADM: Abductor digiti minimi muscle.

### MEP amplitudes

In the static image condition, the independent-samples t-test showed that the MEP amplitude in the FCR muscle did not significantly differ between the expert and novice groups [246.2 ± 41.7 μV in experts and 205.6 ± 28.4 μV in novices, *t* (16) = 0.758, *p* = 0.459]. Similarly, the MEP amplitude in the ADM muscle at the static image condition was not significantly different between the two groups [524.8 ± 60.8 μV in experts and 589.2 ± 124.1 μV in novices, *t* (11.636) = −0.439, *p* = 0.668].

**Fig 2:**
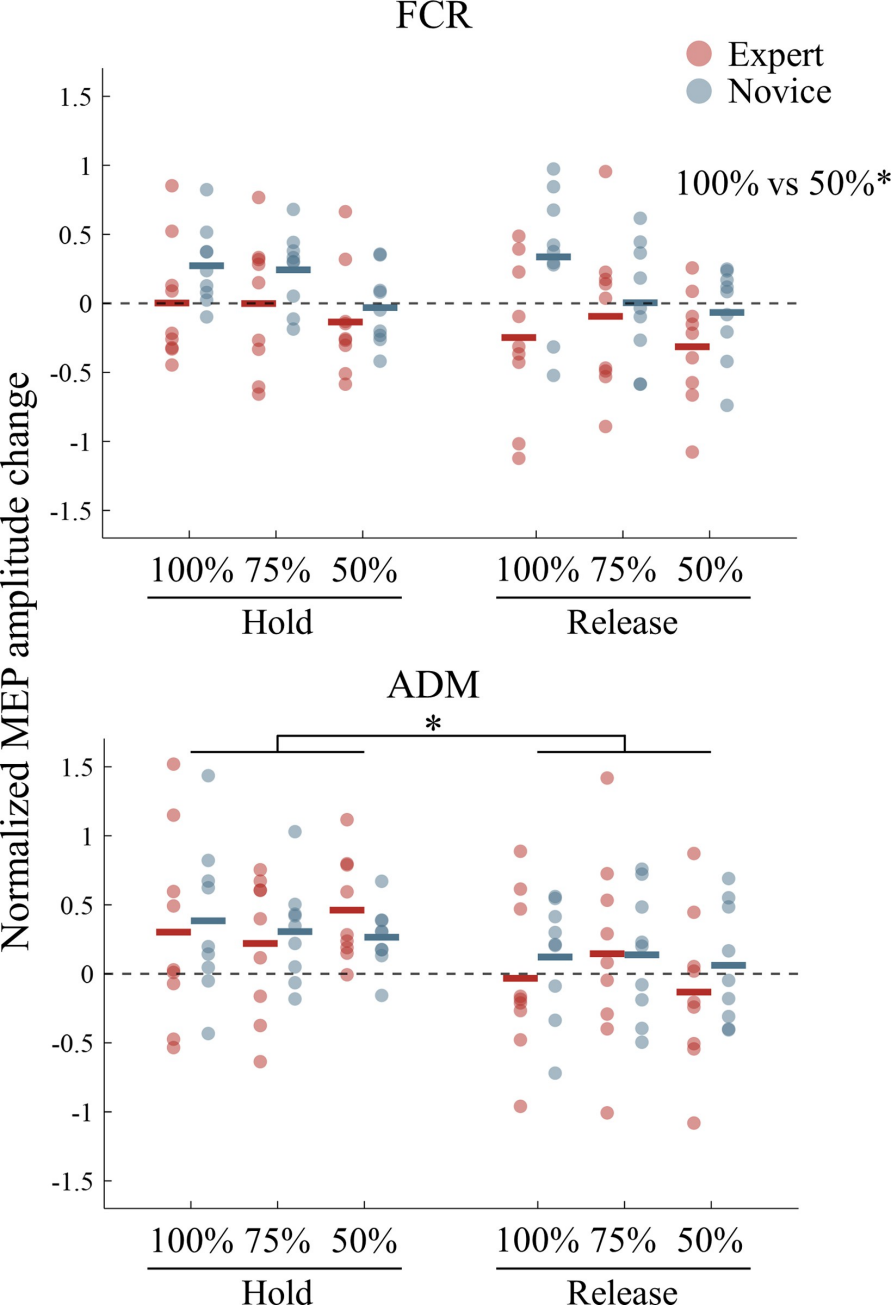
Motor evoked potential (MEP) in each action observation (AO) condition. The upper and lower panels show the z-transformed MEP amplitudes in the flexor carpi radialis (FCR) and abductor digiti minimi (ADM) muscles, respectively. The normalized MEP amplitude in the static image condition was subtracted from that in each AO condition. Value > 0 indicates that the normalized MEP amplitude in the action observation condition was larger than that in the static condition. Red and blue dots indicate the data of the expert and novice groups, respectively. Solid lines represent mean values. *: *p* < 0.05.

The normalized MEP amplitude in each AO condition in each group is shown in Fig 2. In the FCR muscle, analyses using the linear mixed-effects model showed a significant fixed effect of the video playback speed [*F* (2, 80) = 4.835, *p* = 0.010]. As a result of the post-hoc test, the MEP amplitude in the 50% speed condition was significantly smaller than that in the 100% speed condition (*p* = 0.012). In the ADM muscle, a significant fixed effect of stimulus timing was found [*F* (1, 80) = 16.337, *p* < 0.001]. The post-hoc test revealed that the MEP amplitude at Hold timing was significantly larger than that at Release timing. On the other hand, the fixed effect of group and stimulus timing in the FCR muscle [group: *F* (1, 16) = 3.246, *p* = 0.090, stimulus timing: *F* (1, 80) = 3.804, *p* = 0.055], and the fixed effect of group and video playback speed in the ADM muscle [group: *F* (1, 16) = 0.077, *p* = 0.785, video playback speed: *F* (2, 80) = 0.119, *p* = 0.888] were not statistically significant. Moreover, no significant interaction was found in both the FCR [group × stimulus timing: *F* (1, 80) = 0.694, *p* = 0.407, group × video playback speed: *F* (2, 80) = 1.829, *p* = 0.167, stimulus timing × video playback speed, *F* (2, 80) = 0.125, *p* = 0.882, group × stimulus timing × video playback speed: *F* (2, 80) = 1.153, *p* = 0.321] and ADM muscles [group × stimulus timing: *F* (1, 80) = 0.821, *p* = 0.368, group × video playback speed: *F* (2, 80) = 0.270, *p* = 0.764, stimulus timing × video playback speed, *F* (2, 80) = 1.433, *p* = 0.245, group × stimulus timing × video playback speed: *F* (2, 80) = 1.103, *p* = 0.337]. The mean and SD of the raw MEP amplitude in each observation condition for each participant are shown in S1 Table.

## Discussion

A previous study [22] indicated that slowing the video playback speed increased the corticospinal excitability during AO of the ball-catching movement by making the elements of the action easier to recognize. In contrast, our results showed that slowing the video playback speed to 50% reduced the corticospinal excitability of the FCR muscle during AO of a basketball free-throw movement. The difference in the results between the present study and previous studies [22,23] may be due to the complexity of the movements and the visibility of the observed movement. The ball-catching movement presented in the previous studies [22,23] comprised a one-handed finger movement that can be considered a less complex motor action than the basketball free-throw movement with numerous degrees of freedom in both the upper and lower limbs. The slow playback speed during the AO of the free-throw movement may have dispersed the attention and made it difficult to concentrate on the key elements of the action among the many sources of information associated with the action. Concerning the visibility of the movement, previous studies have used video clips from a first-person viewpoint [22,23], while we presented video clips from a third-person perspective. In addition, in the video clips of the present study, since the player, ball, and basketball hoop were within the video frame (as shown in Fig 1), the size of the player visible on the monitor was relatively small. We speculate that the small size of the player and the multiple contexts, including the ball and hoop on the monitor, might have dispersed the observer’s visual attention during the slow playback speed condition. Such dispersion of visual attention owing to the slower playback speed during the AO of the complex motor action may have induced decreased corticospinal excitability. Previously, some reports have shown that the direction of visual attention affects corticospinal excitability during AO [29,30]. For example, in a ball-pinching task, the MEP amplitude did not increase when participants were instructed to observe the video freely compared to the static image condition; however, it did increase when they were instructed to concentrate on the index finger [30]. Therefore, visual attention may need to be directed to body movements for activating the MNS. In the present study, we could not measure the observer’s gaze during AO. Further studies should measure eye movements to detect the dispersion of visual attention at slow playback speeds during AO.

Another possible explanation for the decreased corticospinal excitability at the slower playback speed condition may be the marked speed difference between the observed action and the observer’s own action. Avanzino et al. [24] reported that corticospinal excitability was facilitated during the AO of complex finger motor sequences at 2 Hz in comparison to a static image condition but not during the AO of motor sequences at 1 Hz. The movement tempo at 2 Hz was closest to the spontaneous movement tempo of the participants, indicating that corticospinal excitability during AO is not facilitated when observing slow movements, such as those performed at half the preferred tempo. Therefore, even when observing complex sequential movements involving multiple joints of the lower and upper limbs, such as a basketball free throw, corticospinal excitability might be less facilitated when the action is observed at a considerably slower playback speed than the observer’s actual speed of the same action. Additionally, it has been reported that activity in the motor-related area is affected by movement velocity [31,32]. When observing an actor’s sinusoidal forearm up and down actions, the power of sensorimotor oscillatory activity in the 15–30 Hz range was more attenuated at the period approaching the maximum action speed than that at approaching the minimum action speed [32]. This could be because the attenuation of the sensorimotor oscillatory activity during AO would result from the activation of the MNS in the premotor cortex. Thus, the observations at a slower action speed might have made it difficult to activate the MNS in the present study.

In the FCR muscle, slowing down video playback speed to half of the normal speed significantly decreased corticospinal excitability compared to normal playback speed. On the other hand, the corticospinal excitability of the ADM muscle was not significantly different across the three video playback speed conditions. Therefore, it is considered that slowing down the video playback speed decreases the corticospinal excitability of the FCR muscle during AO of the basketball free-throw movement but did not affect the corticospinal excitability of the ADM. This difference in the effect of video speed on corticospinal excitability between muscles might be explained by the function of muscles during basketball free-throw movement. In basketball free-throw movement, players are advised to spread their fingers almost to the maximum when holding the ball [33]. Thus, the corticospinal excitability of the ADM muscle would be less affected by the video playback speed because the ADM muscle contracts isometrically while holding the ball, with little visible finger movement during the action. On the other hand, the FCR muscle responsible for wrist flexion contracts concentrically to release the ball in the free-throw motion. Therefore, the corticospinal excitability of the muscle involved with visible movement might have been strongly affected by slowing down playback speed for the aforementioned causes.

Regarding the stimulus timing, the normalized MEP amplitude of the ADM muscle during AO of a basketball free-throw movement was larger when holding the ball after dribbling than when releasing it. These results indicate that the corticospinal excitability of the ADM muscle during AO of free-throw movement is modulated depending on the phase of the movement. It has been widely reported that observing others’ action increases the corticospinal excitability of muscles involved during the execution of that action [14] and that the corticospinal excitability during AO is more facilitated at the movement phase in which the muscle would be activated. [20,21]. Since the ADM muscle would be activated when spreading the little finger to hold a ball after dribbling during free-throw motion, the corticospinal excitability of the ADM muscle might have temporarily increased when observing the player in the video spreading the finger to hold the ball. On the other hand, the normalized MEP amplitude of the FCR was not significantly different between the stimulus timings. The wrist flexion was also used during dribbling just before ball holding, which may have resulted in no difference between stimulus timings in the corticospinal excitability of the FCR.

In the context of motor expertise, a previous functional magnetic resonance imaging (fMRI) study showed that the classical mirror areas, including the premotor cortex, parietal cortex, and superior temporal sulcus, are activated more when observing a dance action that was in the observer’s motor repertoire than when it was not in their repertoire [25]. Therefore, we hypothesized that basketball experts may activate the premotor area more than novices when observing a basketball free-throw movement and that corticospinal excitability would be much more facilitated by communication between the premotor area and M1 [34,35] in the experts than in the novices. However, our results demonstrated no significant differences of the MEP amplitude between experts and novices during the free-throw observation. A previous report by Aglioti et al. [27] also showed that the MEP amplitudes of the ADM and flexor carpi ulnaris muscles were increased in both experts and novices during the observation of basketball free-throw movement. Additionally, they reported that novices also facilitate the corticospinal excitability of the corresponding upper limb muscles when observing a soccer kick movement. Thus, they suggested that the observation of body movements by novices leads to nonspecific activation of the motor system. Considering these findings, motor expertise might not affect corticospinal excitability when observing dynamic movement.

AO intervention might contribute to improvement in motor performance, at least partially by functional plasticity of motor-related brain areas [5,6]. Our results suggest that slowing the video speed would be less effective in facilitating corticospinal excitability during the AO of complex whole-body movements. The effectiveness of utilizing slow playback speed videos as a practice method of basketball free-throw movement was not demonstrated from a neurophysiological aspect. Future studies should verify detailed brain activity during the observation of slow playback speed video using fMRI to investigate the relevance of the MNS. Comparing brain activity during observation at normal and slow video playback speeds would reveal how the recognition of observed action change at slow playback speed affects the brain activity.

## Conclusion

The novel finding of this study was that the corticospinal excitability of the FCR muscle was decreased when the video playback speed was decreased to half of the normal speed during the observation of a basketball free-throw movement. On the other hand, the corticospinal excitability of the ADM muscle was modulated depending on the phase of the movement. Additionally, motor expertise did not affect corticospinal excitability during the observation of free-throw movement. These study findings may provide information to deepen our understanding of the neural mechanisms underlying AO.

## Supporting information

S1 Table Mean and standard deviation of background electromyographic activity and motor evoked potential amplitude in each observation condition for each participant. (PDF)
