# Supplementary material for: Corticospinal excitability during observation of basketball free-throw movement: Effects of video playback speed and stimulus timing
Source: PLoS One. 2023 Sep 28;18(9):e0292060. doi: 10.1371/journal.pone.0292060 (PMC10538764; doi:10.1371/journal.pone.0292060)
Supplement: S1 Table — (PDF) [file pone.0292060.s001.pdf]

ID	Group	Background electromyographic activity (μV, Mean ± standard deviation)													
		FCR							ADM						
		Static	Hold			Release			Static	Hold			Release		
			100%	75%	50%	100%	75%	50%		100%	75%	50%	100%	75%	50%
1	Expert	1.62 ± 0.59	1.83 ± 0.81	1.76 ± 0.65	2.09 ± 0.76	1.63 ± 0.82	1.61 ± 0.84	1.61 ± 0.52	5.05 ± 1.84	4.17 ± 1.44	4.44 ± 1.84	4.73 ± 1.59	3.23 ± 1.83	3.08 ± 1.22	3.12 ± 1.82
2	Expert	5.53 ± 2.37	4.81 ± 1.24	4.66 ± 1.21	4.92 ± 2.58	5.87 ± 2.48	5.23 ± 2.36	4.90 ± 1.71	2.76 ± 1.21	3.32 ± 2.26	2.76 ± 1.79	4.19 ± 2.25	3.07 ± 1.89	2.74 ± 1.12	2.17 ± 1.40
3	Expert	5.25 ± 0.63	5.34 ± 0.56	5.09 ± 0.69	5.24 ± 1.24	4.79 ± 0.99	4.96 ± 0.82	4.82 ± 1.12	7.07 ± 1.56	8.14 ± 0.89	7.18 ± 0.78	7.06 ± 1.03	5.73 ± 1.66	7.13 ± 1.58	5.39 ± 1.53
4	Expert	2.75 ± 0.80	2.94 ± 0.76	2.73 ± 0.65	2.61 ± 1.08	2.73 ± 0.74	2.54 ± 1.08	2.10 ± 1.02	3.65 ± 3.18	4.79 ± 1.75	3.46 ± 1.77	5.01 ± 1.77	5.02 ± 2.89	4.63 ± 1.83	4.45 ± 2.96
5	Expert	3.66 ± 0.97	4.27 ± 2.07	3.63 ± 0.82	2.92 ± 0.82	3.48 ± 1.75	3.64 ± 1.74	3.01 ± 0.75	4.01 ± 1.39	3.64 ± 1.17	3.64 ± 1.72	3.60 ± 1.71	3.28 ± 0.92	2.89 ± 1.00	3.56 ± 1.55
6	Expert	2.46 ± 0.72	2.75 ± 1.08	2.33 ± 0.61	3.32 ± 1.35	2.76 ± 0.85	3.77 ± 1.87	2.78 ± 0.92	2.36 ± 0.61	3.10 ± 0.69	2.55 ± 0.88	2.49 ± 0.88	3.03 ± 1.14	3.00 ± 1.14	2.42 ± 0.67
7	Expert	3.40 ± 1.11	2.81 ± 1.03	2.84 ± 0.92	2.50 ± 0.60	2.94 ± 0.88	3.10 ± 1.13	2.86 ± 0.84	3.62 ± 0.80	3.35 ± 1.35	3.97 ± 1.27	4.38 ± 0.89	4.61 ± 0.92	4.15 ± 1.54	4.62 ± 1.19
8	Expert	2.23 ± 1.04	1.89 ± 0.87	1.79 ± 0.56	1.85 ± 0.65	2.13 ± 0.99	1.98 ± 1.04	1.85 ± 0.72	2.86 ± 1.52	3.55 ± 1.89	3.65 ± 1.79	3.00 ± 1.14	2.16 ± 0.82	2.50 ± 1.63	1.73 ± 0.60
9	Expert	2.76 ± 0.90	2.78 ± 0.71	2.87 ± 0.53	3.01 ± 1.03	3.41 ± 1.30	3.43 ± 0.75	2.92 ± 1.09	2.31 ± 0.54	2.47 ± 0.69	2.58 ± 0.81	3.16 ± 1.18	2.76 ± 0.96	2.69 ± 0.62	2.46 ± 0.87
10	Novice	4.34 ± 1.35	4.22 ± 1.29	3.73 ± 0.98	2.80 ± 1.40	3.26 ± 1.41	3.64 ± 1.41	2.16 ± 0.89	3.35 ± 1.98	4.11 ± 2.08	3.38 ± 2.81	3.26 ± 1.90	3.75 ± 2.33	3.16 ± 1.79	3.52 ± 1.85
11	Novice	3.22 ± 1.29	2.96 ± 0.86	2.53 ± 0.90	2.08 ± 0.54	2.94 ± 1.21	2.56 ± 1.07	2.98 ± 1.12	1.80 ± 0.60	1.93 ± 0.89	2.92 ± 1.41	1.72 ± 0.87	2.11 ± 1.23	1.87 ± 0.62	2.40 ± 1.02
12	Novice	3.00 ± 1.28	2.83 ± 0.57	3.57 ± 1.19	3.42 ± 1.40	2.61 ± 0.56	3.06 ± 0.52	3.21 ± 1.47	2.76 ± 1.44	3.58 ± 1.41	2.96 ± 1.25	3.15 ± 1.63	2.99 ± 1.36	3.34 ± 1.70	2.67 ± 1.53
13	Novice	2.68 ± 1.21	2.62 ± 1.05	2.49 ± 1.13	2.23 ± 1.34	3.23 ± 1.57	2.34 ± 1.56	2.81 ± 1.56	3.62 ± 1.45	4.27 ± 1.82	4.18 ± 1.75	3.76 ± 1.35	4.05 ± 1.74	4.05 ± 1.06	4.55 ± 1.76
14	Novice	3.05 ± 0.78	2.94 ± 0.64	3.13 ± 0.72	2.75 ± 0.75	3.04 ± 0.60	2.55 ± 0.63	2.52 ± 0.49	2.99 ± 1.13	3.35 ± 1.47	2.74 ± 0.98	2.72 ± 0.85	2.57 ± 0.60	3.21 ± 1.29	3.14 ± 1.14
15	Novice	4.14 ± 1.19	3.97 ± 1.76	4.91 ± 1.51	3.86 ± 0.99	4.93 ± 1.78	3.12 ± 0.84	4.33 ± 1.95	3.91 ± 1.71	4.41 ± 1.31	4.02 ± 1.59	3.52 ± 1.12	3.42 ± 1.27	3.80 ± 0.74	3.37 ± 1.92
16	Novice	2.29 ± 0.57	2.43 ± 0.55	2.41 ± 0.67	2.41 ± 0.45	2.67 ± 0.48	2.27 ± 0.47	2.78 ± 0.61	1.75 ± 0.44	1.97 ± 0.87	1.79 ± 0.43	1.61 ± 0.44	1.55 ± 0.25	1.70 ± 0.51	1.73 ± 0.40
17	Novice	2.53 ± 0.73	2.35 ± 0.83	2.59 ± 0.75	2.65 ± 0.66	2.71 ± 0.61	2.37 ± 0.60	2.70 ± 1.00	2.25 ± 1.09	2.79 ± 1.05	2.57 ± 0.86	2.07 ± 0.75	2.47 ± 0.57	2.28 ± 0.64	2.60 ± 1.12
18	Novice	3.45 ± 1.33	2.71 ± 0.60	3.04 ± 1.34	3.40 ± 0.96	3.03 ± 0.94	3.04 ± 0.97	3.10 ± 0.90	4.35 ± 1.40	4.04 ± 1.37	4.89 ± 1.12	5.71 ± 1.91	5.29 ± 3.11	3.10 ± 1.30	5.54 ± 2.02

FCR: flexor carpi radialis muscle; ADM: abductor digiti minimi muscle

ID	Group	Motor evoked potential amplitude (μV, Mean ± standard deviation)													
		FCR							ADM						
		Static	Hold			Release			Static	Hold			Release		
			100%	75%	50%	100%	75%	50%		100%	75%	50%	100%	75%	50%
1	Expert	336.4 ± 112.4	301.8 ± 107.7	308.4 ± 92.2	275.1 ± 75.0	229.9 ± 96.8	280.8 ± 122.1	223.7 ± 52.7	726.4 ± 211.8	593.6 ± 169.3	633.3 ± 279.0	724.8 ± 188.5	487.7 ± 264.4	476.0 ± 181.1	457.7 ± 255.5
2	Expert	535.4 ± 275.6	569.8 ± 288.7	447.5 ± 324.2	464.9 ± 291.7	510.4 ± 228.7	299.5 ± 128.3	383.5 ± 135.4	809.3 ± 321.5	1030.6 ± 334.0	988.8 ± 556.2	1076.7 ± 414.5	1085.5 ± 489.3	1049.2 ± 446.9	701.0 ± 389.2
3	Expert	180.9 ± 31.9	169.9 ± 37.8	160.3 ± 37.0	170.5 ± 37.7	142.7 ± 27.2	164.9 ± 24.5	167.5 ± 26.0	599.1 ± 88.4	590.0 ± 122.5	517.0 ± 98.0	743.1 ± 81.8	537.4 ± 166.7	561.5 ± 120.8	533.9 ± 55.3
4	Expert	320.7 ± 104.6	285.1 ± 67.2	268.3 ± 62.8	280.1 ± 76.6	291.5 ± 70.5	338.8 ± 51.2	303.5 ± 88.8	514.1 ± 115.6	1065.7 ± 318.0	787.7 ± 191.1	800.4 ± 350.3	684.7 ± 301.5	777.5 ± 454.1	830.5 ± 415.5
5	Expert	227.0 ± 54.2	202.1 ± 50.5	254.1 ± 111.0	212.9 ± 109.7	186.1 ± 123.8	180.0 ± 59.8	235.4 ± 106.8	607.6 ± 197.7	513.0 ± 143.8	630.7 ± 212.5	664.1 ± 215.6	575.1 ± 174.4	527.9 ± 200.1	566.4 ± 199.5
6	Expert	163.6 ± 44.6	245.9 ± 106.6	195.6 ± 83.4	227.7 ± 105.4	210.6 ± 88.9	255.8 ± 95.2	188.4 ± 101.5	204.6 ± 42.9	325.2 ± 127.5	275.1 ± 121.2	288.3 ± 58.7	297.8 ± 102.6	353.4 ± 83.3	251.4 ± 96.0
7	Expert	188.5 ± 123.1	198.2 ± 85.6	204.6 ± 94.6	160.8 ± 61.7	154.7 ± 109.6	207.2 ± 137.2	116.9 ± 92.1	285.8 ± 116.3	289.0 ± 104.2	267.8 ± 135.2	302.4 ± 87.3	265.4 ± 108.4	294.9 ± 102.8	288.0 ± 111.0
8	Expert	95.9 ± 38.6	115.1 ± 24.9	124.0 ± 41.6	107.6 ± 24.3	104.3 ± 46.9	97.3 ± 34.5	90.4 ± 26.4	529.6 ± 322.8	705.0 ± 301.4	707.6 ± 297.9	585.5 ± 242.3	451.2 ± 248.5	516.1 ± 277.0	369.7 ± 175.4
9	Expert	167.7 ± 31.7	160.4 ± 34.2	178.4 ± 39.5	163.3 ± 35.9	181.0 ± 23.3	172.5 ± 33.6	164.5 ± 30.5	446.4 ± 224.1	449.4 ± 274.0	618.3 ± 296.4	513.6 ± 350.6	386.6 ± 252.1	528.6 ± 281.3	461.4 ± 220.9
10	Novice	325.6 ± 51.0	337.2 ± 97.1	353.2 ± 131.9	357.6 ± 57.5	277.8 ± 74.1	272.2 ± 87.1	258.0 ± 49.9	1032.3 ± 210.6	1014.6 ± 221.0	1049.8 ± 466.1	1167.9 ± 395.1	1001.5 ± 414.6	1004.9 ± 361.2	891.0 ± 221.3
11	Novice	229.2 ± 77.6	222.3 ± 54.4	250.2 ± 67.5	199.8 ± 43.2	248.8 ± 100.9	226.8 ± 52.4	245.7 ± 64.1	432.5 ± 176.0	440.4 ± 142.2	491.9 ± 198.2	405.7 ± 115.0	467.7 ± 203.6	400.3 ± 119.7	461.0 ± 195.9
12	Novice	106.7 ± 33.5	136.5 ± 42.8	122.7 ± 30.4	97.2 ± 19.4	120.3 ± 37.2	129.0 ± 39.8	111.0 ± 28.5	398.2 ± 181.3	522.9 ± 226.8	439.0 ± 133.2	422.7 ± 163.8	453.7 ± 197.2	532.2 ± 151.1	324.1 ± 138.1
13	Novice	305.3 ± 120.6	336.4 ± 158.0	355.0 ± 117.0	279.0 ± 100.5	343.8 ± 136.3	353.1 ± 145.4	316.5 ± 108.4	1034.4 ± 409.7	1099.1 ± 373.9	1224.8 ± 552.5	1114.2 ± 344.8	1130.8 ± 556.1	1137.8 ± 413.2	1254.0 ± 436.0
14	Novice	223.1 ± 69.3	256.0 ± 103.5	227.7 ± 76.9	254.5 ± 73.0	195.4 ± 100.8	199.7 ± 93.6	186.2 ± 59.8	606.8 ± 187.1	764.0 ± 307.3	733.6 ± 212.5	775.7 ± 218.5	521.9 ± 255.5	657.3 ± 273.9	561.5 ± 137.6
15	Novice	75.5 ± 35.5	76.7 ± 34.2	70.2 ± 26.2	80.0 ± 36.3	121.4 ± 80.2	47.9 ± 27.5	65.8 ± 20.7	264.9 ± 171.5	302.9 ± 142.6	252.5 ± 226.3	324.2 ± 245.0	126.2 ± 92.0	188.6 ± 162.0	205.2 ± 184.8
16	Novice	162.5 ± 40.3	176.0 ± 29.4	187.3 ± 35.4	165.4 ± 26.0	177.8 ± 16.8	169.1 ± 47.3	168.6 ± 42.7	237.3 ± 125.8	439.9 ± 148.7	382.7 ± 127.4	280.3 ± 130.5	295.8 ± 106.4	305.6 ± 130.6	315.1 ± 117.5
17	Novice	135.0 ± 59.6	175.3 ± 93.5	120.4 ± 77.5	116.9 ± 69.0	187.8 ± 84.3	127.4 ± 49.0	154.4 ± 73.9	121.1 ± 60.1	175.7 ± 71.0	109.0 ± 44.9	132.6 ± 57.9	157.4 ± 77.8	171.5 ± 30.1	167.0 ± 77.8
18	Novice	287.8 ± 131.4	298.4 ± 161.6	332.2 ± 114.7	281.4 ± 104.8	400.9 ± 139.6	347.3 ± 112.4	276.9 ± 117.5	1175.4 ± 391.9	1013.3 ± 358.0	1337.4 ± 284.4	1320.1 ± 342.7	1385.3 ± 361.3	989.8 ± 302.5	1157.7 ± 347.8

FCR: flexor carpi radialis muscle; ADM: abductor digiti minimi muscle
